# Health equity and the global social contract: beyond incrementalism and illusionary solidarity

**DOI:** 10.1186/s12939-026-02773-7

**Published:** 2026-02-21

**Authors:** Moses Mulumba, Jessica Oga, Christopher Baguma, Juliana Nantaba, Ana Lorena Ruano

**Affiliations:** 1 Afya na Haki Institute, Nakwero, Uganda; 2 Impacting Health Hub, Liverpool, UK; 3 Centre for International Health, University of Bergen, Bergen, Norway

**Keywords:** Global health governance, Global social contract, Health equity, Reparative justice, Sustainable development goals

## Abstract

The Millennium Development Goals (MDGs) and Sustainable Development Goals (SDGs) have been celebrated as global social contracts, yet their reliance on voluntary commitments and aspirational targets conceals a structural flaw. By divorcing poverty and inequity from colonial histories, debt regimes, and extractive global finance, these frameworks function as a neocolonial placebo: soothing global conscience while entrenching asymmetries of power and resources. Drawing on examples from debt distress, vaccine apartheid, and intellectual property monopolies during COVID-19, this commentary demonstrates that global health governance operates less as solidarity than as economic containment. Reparative justice provides the necessary rupture. A post-2030 Global Social Contract must impose enforceable obligations on former colonial powers, embed structural restitution through debt and tax justice, and democratise health governance under the principle of Common but Differentiated Responsibilities. Anything less risks reproducing selective generosity while abandoning equity to the logics of extraction and impunity.

## Introduction

The Millennium Development Goals (MDGs) and their successor, the Sustainable Development Goals (SDGs), represent the closest approximation to a Global Social Contract (GSC) in modern history, a consensus framework in which nations pledged to eradicate poverty, reduce inequality, and advance health equity.^1^ Yet, as donor states slash Official Development Assistance (ODA) budgets to prioritise geopolitical or trade-aligned priorities, the fragility of this contract and its consequences for global health equity are laid bare.^2^

These global goals rely on voluntary commitments and non-binding targets, eschewing enforceable obligations to redress the structural violence of colonialism, debt apartheid, and extractive fiscal policies.^3^ This is not merely a failure of implementation but a deeper failure of design. The framework is embedded in an international order organised around state sovereignty and voluntary cooperation, which international relations theorists term “anarchy”.^4^ In the absence of a central authority capable of enforcing binding obligations, global commitments inevitably default to aspirational targets and donor discretion. This systemic structural flow ensures that colonial legacies and structural exploitation remain unaddressed, while asymmetries of power, capital, and voice are preserved beneath the rhetoric of solidarity.

The consequences are stark. In 2023, thirty-two African countries allocated more on debt servicing than on healthcare, while high-income countries contributed just 0.2% of gross national income (about USD 120 billion) to ODA^5^- a figure dwarfed by the USD 1 trillion annually drained from the Global South through illicit financial flows, regressive tax regimes, and pharmaceutical monopolies.^6^ The SDGs’ emphasis on “partnerships” and “shared responsibility” obscures the reality that contemporary global health governance operates as a neocolonial project, relegating low- and middle-income countries (LMICs) to pleading for charity while wealthy nations evade accountability for historical and ongoing harm.^7^ In 2020, President Trump’s demand for pandemic reparations from China, though politically fraught, inadvertently exposed what the SDG framework refuses to confront: *systemic harms require systemic redress, not rhetorical appeals to solidarity*.^8^

As the world moves toward the post-2030 development agenda, this commentary argues for a new Global Social Contract that abandons the technocratic optimism of the SDG framework and is instead rooted in the political and legal imperative of reparative justice. Methodologically, the analysis is normative and doctrinal. It engages global health governance frameworks (such as the MDGs, SDGs, and TRIPS), international economic law, and critical theoretical traditions to synthesise historical precedents with contemporary policy failures. The aim is not empirical measurement but conceptual reframing: to position a reparative Global Social Contract as the necessary rupture with the SDG paradigm.

## The MDG-SDG continuum: a social contract of convenience, not justice

The MDGs and SDGs emerged as moral compacts, yet their architectures reveal a deliberate evasion of historical accountability.^9^ The MDGs, framed in 2000 as a “blueprint to end poverty”, ignored the colonial roots of poverty, instead attributing inequity to poor governance and resource scarcity in LMICs, absolving high-income states of their colonial and extractive legacies.^10^ The SDGs, while nominally addressing “structural barriers”, replicated this omission, reducing reparative justice to vague calls for “policy coherence” (Goal 17) and “reducing inequalities” (Goal 10) devoid of binding mechanisms. This omission was not incidental but strategic: by divorcing poverty from its colonial roots, the MDG–SDG framework enabled donor states to posture as allies while perpetuating the very systems that impoverish nations: Structural Adjustment Programs (SAPs),^11^ Agreement on Trade-Related Aspects of Intellectual Property Right (TRIPS)-plus, and austerity mandates.

The SDGs’ failures are not accidents of implementation but products of design. The SDG Target 3.b for instance, which pledges to “support research and development of vaccines and medicines for all,” failed catastrophically during COVID-19 under the weight of neocolonial profiteering.^12^ High-income countries representing 16% of the global population, hoarded 70% of vaccine doses by mid-2021, while sub-Saharan Africa received less than 1%.^13^ The European Union (EU) threatened India with export bans to prevent generic vaccine production, and pharmaceutical giants like Pfizer leveraged TRIPS-plus clauses to deny LMICs access to mRNA technologies.^14^ Meanwhile, The World Trade Organisation (WTO) spent 18 months debating and ultimately diluting a TRIPS waiver, ensuring medicines remained luxury commodities.^15^ The result was a “death gap”: LMICs experienced COVID-19 mortality rates 3.5 times higher than high-income states, revealing how SDG-framed “solidarity” collapses under the weight of profit logic.^16^

Equally illusory is Target 17.4, which promises to “help developing countries achieve debt sustainability”, even as debt servicing accelerates across the Global South. As of 2024, about 54% of LMICs were in or at high risk of debt distress -a sharp deterioration from the mid-2010s.^17^ At least 46 developing countries now spend more on interest payments than on either health or education, affecting roughly 3.4 billion people.^18^ The burden falls disproportionately on Africa: between 2019 and 2021, 25 African countries -nearly half the continent spend more on interest than on health, underscoring how interest costs are crowding out social expenditure.^19^ Regionally. UN system analyses also found that developing countries in Asia and Oceania (excluding China) are allocating more funds to interest payments than to health, while Latin America and the Caribbean, allocate more to interest payments than to public investment.^20^

Several countries face especially acute squeezes: recent compilations show include Gambia, Ghana, Zambia, Laos, Lebanon and Pakistan, among those spending over 20% of government revenue on external debt payments. Zambia illustrates the trade-offs: debt repayments rose from 20% to 38% of the national budget (2018–2021) as the health share fell from 9.5% (2018) to 8% (2022). Following its IMF arrangement, energy-price reforms and subsidy removals increased fuel and electricity prices, tightening household welfatr and fiscal space debated as “austerity” in local assessments.

The SDG framework offers no mechanism to identify or remedy “odious debt”, even in the face of well-documented cases of predatory lending built on undisclosed state guarantees.^21^ The most striking example is Mozambique’s $2.7 billion “tuna bond” scandal,^22^ a fraudulent loan scheme orchestrated through Credit Suisse and Privinvest that diverted funds ostensibly intended for fisheries and maritime security into private enrichment and political kickbacks. Despite the magnitude of this financial crime and its devastating consequences for public debt and social spending, the SDGs remain conspicuously silent, providing no framework for accountability or relief in such cases.

These outcomes reflect the MDG-SDG continuum’s core contradiction, that *it conflates justice with charity*. The World Bank’s “Maximising Finance for Development” strategy—lauded under SDG 17, for example, forces LMICs to replace public health funding with public-private partnerships (PPPs), breaking systems into profit-driven fragments.^23^ This replicates the structural adjustment playbook of the 1980s, which slashed public health spending in 36 African nations by 50%, precipitating a collapse in life expectancy.^24^

The SDGs’ voluntary framework allows powerful states to manipulate international aid for political ends.^25^ In January 2025, the United States implemented significant cuts to the President’s Emergency Plan for AIDS Relief (PEPFAR), disrupting HIV/AIDS programs across Africa and disproportionately affecting LGBTQ+ communities.^26^ Simultaneously, the UK redirected approximately 28% of its aid budget to cover domestic asylum-related expenses, diverting funds from international development initiatives.^27^ These actions underscore the fragility of the SDGs when confronted with donor nations’ political self-interest.

Ultimately, the SDGs operate as a neocolonial placebo -soothing global conscience without treating the root causes of injustice. By refusing to name colonialism, slavery, and capitalist extraction as foundational to global inequality, the framework renders reparative justice unthinkable. Fanon’s reflections remain uncannily resonant here. Writing in *The Wretched of the Earth*, he rejected the framing of development assistance as charity, insisting that what is portrayed as aid must be understood as reparations owed.^28^ For Fanon, such“help”could never be the benevolent gesture of former colonial powers but rather the recognition of a historical debt – “a reparation which will be paid to us”.

Ghana’s $65 billion loss from British gold extraction or Indonesia’s $165 billion plunder by the Dutch remains unacknowledged, while donor states applaud ODA flows that stagnate at 0.3% of GNI.^29^ Unless the post-2030 development agenda reconfigures the GSC to ensure legal accountability and reparations, global health will remain a theatre of selective generosity rather than a platform for equity and justice.

## Global health governance as an instrument of economic control: the structural limits of sovereignty for LMICs

The MDG-SDG framework’s failure is not an anomaly but an extension of a colonial logic that has long conflated health security with capital protection.^30^ The 1892 International Sanitary Convention, designed to safeguard European trade from cholera outbreaks, established a precedent where disease containment prioritised commerce over care—a paradigm that persists today.^31^ Postcolonial institutions like the WHO inherited this legacy, as high-income states weaponised funding to constrain LMIC sovereignty.^32^ For instance, the USA withheld dues to the WHO in the 1980s to block its support for generic drug production, ensuring pharmaceutical monopolies remained intact.^33^ This historical continuum reveals global health not as a rupture from colonialism but its evolution, where ostensibly neutral frameworks like the International Health Regulations (2005) still prioritise economic stability over equitable access.^34^

Contemporary health financing replicates these asymmetries through regimes of debt and austerity. Structural adjustment programmes (SAPs) imposed by the International Monetary Fund in the 1980s forced LMICs to slash health budgets, privatise care, and adopt user fees—policies that precipitated a 50% decline in public health spending across sub-Saharan Africa, institutionalising inequities by privatising care and shifting costs to impoverished households, thereby excluding the poorest from accessing essential services.^35^ This dependency cycle entrenches a form of “predatory global health,” where concessional loans and public-private partnerships bind LMICs to donor agendas. In Ghana, successive IMF programmes imposed austerity measures and regressive taxation, resulting in cuts to social spending, particularly in health, while increasing shares of national revenue were diverted into debt servicing.^36^

Legal architectures further codify this subordination. The WTO’s TRIPS Agreement granting 20-year drug patents enabled pharmaceutical firms to sue South Africa in 1998 for producing generic HIV/AIDS medicines, a move the international community, including the WHO, condemned as “a violation of the right to health”.^37^ Despite the Doha Declaration’s flexibilities, LMICs continue to face coercion to abandon compulsory licensing. During COVID-19, negotiations at the World Trade Organization over a TRIPS waiver dragged on for 18 months, only to be diluted under pressure from the EU and other high-income states, ensuring that lifesaving technologies remained monopolised.^38^ These barriers are not technical but political, ensuring LMICs remain consumers rather than producers in a health ecosystem where the majority of vaccine R&D is concentrated in high-income states.^39^ Until good governance dismantles these hierarchies, global health will remain a system in which access to life-saving resources is determined not by need but by economic hierarchies that limit LMICs’ ability to exercise health sovereignty.

## Operationalising a global social contract

Global health governance is characterised by a fundamental contradiction: it rhetorically commits to advancing equity while structurally reproducing the economic hierarchies responsible for the inequities it seeks to remedy. Persisting disparities in avoidable mortality, asymmetrical access to essential medicines, and the fiscal subjugation of low- and middle-income countries are not a failure of intent, but a systemic consequence of governance architectures designed to prioritise profit and geopolitical control over the realisation of health justice.^40^ Addressing these entrenched inequities necessitates the institutionalisation of a reparative logic that transforms global health governance from a vehicle of economic containment into a juridical framework for justice. This transformation must begin by displacing the discretionary architecture of aid with binding obligations to redress the historical and structural harms that continue to define the global health landscape.

At its foundation, the Global Social Contract must reconstitute financing not as a function of benevolence, but of restitution. Unlike Official Development Assistance, which operates through donor discretion and conditionalities, reparative health financing must be governed by obligations anchored in historical accountability.^41^ This call for restitution has historical antecedents that remain instructive. The 1978 Declaration of Alma-Ata explicitly linked health equity to the demand for a New International Economic Order (NIEO), recognising that global health justice required economic restructuring. Similarly, the United Nations General Assembly’s Declaration on the NIEO affirmed“the right of all States, territories and peoples under foreign occupation, alien and colonial domination or apartheid to restitution and full compensation for the exploitation and depletion of, and damages to, their natural resources and all other resources.”These texts, though politically sidelined, demonstrate that reparative logics were once central to international discourse. Re-engaging these traditions not only legitimises but strengthens the normative foundations for a binding, reparative Global Social Contract.

Historical precedents, such as the Jubilee 2000 movement debt cancellation, illustrate the feasibility of large-scale fiscal reparations.^42^ Yet the GSC must go further by establishing a permanent system of financial restitution through global tax justice regimes, annulment of odious debts, and a 0.1% financial transaction levy on high-income states to fund health system recovery in formerly colonised regions. These resources would not be channelled through donor-controlled agencies but rather through a Global Health Equity Fund, with allocations determined by LMIC-led priorities and governance vested in a board composed of LMIC institutions, patient groups, professional organisations, grassroots movements, and civil society organisations.

The operationalisation of the fund must be hinged within a legally binding framework: a United Nations-mandated Framework Convention on Global Health Reparations,^43^ that would codify differentiated responsibilities according to each state’s historical contribution to global health inequities. Modelled on the precedent of the Framework Convention on Climate Change,^44^ the treaty would impose enforceable obligations, departing from the voluntary architecture of the SDGs. Central to its enforcement would be a **Global Health Reparations Tribunal**, a quasi-judicial body empowered to receive claims, adjudicate violations, and mandate restitution for quantifiable health and economic harms resulting from colonial plunder, pharmaceutical exclusion, and austerity-induced health crises.

Within this framework, intellectual property regimes would be restructured through treaty provisions mandating automatic TRIPS waivers for essential medicines, binding technology transfer, and the decommodification of lifesaving innovations. These obligations would no longer depend on the goodwill of pharmaceutical firms or the compromise of WTO negotiations. They would instead be codified as non-derogable health rights, enforceable under international law.

This is not a utopian vision—it is a structural imperative. Without enforceable redress, the post-2030 development agenda will merely recycle the inequities of the past under new technocratic language. The GSC proposes a rupture: one that shifts the locus of global health governance from donor capitals to sites of historical injury; from discretionary generosity to legal obligation; from global health as charity to global health as reparations. It affirms that the right to health cannot be fulfilled until the conditions that systematically undermine it—extraction, exclusion, and impunity—are dismantled at their root.

## Conclusion

What must follow the collapse of the SDG consensus is not another cycle of aspirational targets but a rupture that binds global health to justice. A reparative Global Social Contract offers that framework. It would impose enforceable obligations on former colonial powers and high-income states to confront the legacies of slavery, plunder, and dispossession; embed structural restitution through debt justice, recovery of illicit financial flows, and fair access to medicines and technologies; and democratise global health governance by anchoring authority in the leadership of low- and middle-income countries under the principle of Common but Differentiated Responsibilities. These are not gestures of reform but conditions of justice, the minimum terms upon which any future of global health equity can stand.

## Data Availability

No datasets were generated or analysed during the current study.

## Footnotes

1 Piper and Walsham [1]

2 Moses Mulumba and others [2] See also, Nelson Aghogho Evaborhene and Jessica Oga [3]

3 Susan Forde et al. [4] See also, Mary Menton [5] See also Patrick Bond [6]

4 Kenneth Oye [7].

5 Debt Justice [8] See also, United Nations [9] See also, OECD [10]

6 Debt Justice, ‘The Colonial Roots of Global South Debt: A Tale of Plunder, Exploitation and Resistance’, https://debtjustice.org.uk/wp-content/uploads/2023/09/The-colonial-roots-of-global-south-debt.pdf accessed 19 March 2025. See also, David Lawder, ‘Developing Countries’ Record $1.4 Trillion Debt Service Bill Squeezes Budgets’ Reuters (3 December 2024) https://www.reuters.com/markets/developing-countries-record-14-trillion-debt-service-bill-squeezes-budgets-2024–12-03/ accessed 17 March 2025. See also, African Union [11]

7 Seyi-Olajide [12]

8 Jason Lemon [13]

9 CPB [14]

10 Paul Nelson [15] See also, Donald and Way [16]

11 Stephen McCloskey [17]

12 Archisha Satyarthi [18]

13 WHO [19] See also, Tedros Adhanom Ghebreyesus, ‘Dose of Reality: How rich countries and pharmaceutical corporations are breaking their vaccine promises’, https://www.webassets.oxfamamerica.org/media/documents/A_Dose_of_Reality-Briefing_Note_kOW1yUs.pdf?utm_source= accessed 14 April 2025.

14 Médecins Sans Frontières Access Campaign [20] See also, Médecins Sans Frontières Access Campaign [21]

15 Tahir Amin and Aaron S Kesselheim [22], accessed 4 January 2023.

16 Levin [23] See also, Anwar Khedr and others [24] See also, Nicola Davis [25]

17 Marianna Lunardini [26]

18 UNCTAD [27] See also, CJ Polychroniou [28], https://www.globalpolicyjournal.com/blog/18/12/2024/global-south-brink-disastrous-debt-crisis-reform-urgent?utm_source accessed 14 April 2025. See also, UNCTAD ‘A World of Debt 2024’ (UN Trade and Development (UNCTAD)4 June 2024) https://www.unctad.org/publication/world-of-debt?utm_source accessed 14 April 2025.

19 UNCTAD, supra note 19.

20 Al Jazeera [29]

21 ITUC CSI [30]

22 Reuters Staff [31]

23 Luiz Vieira [32]

24 University of Pennsylvania - African Studies Center [33]

25 Bexell and Jonsson [34]

26 Kat Lay [35] See also UNAIDS [36]

27 ICAI [37]

28 Frantz Fanon [38]

29 James Tuffour, Swetketu Patnaik and Maureen Ayikoru [39] See also, Alec Gordon [40]

30 Clare Wenham, Joshua Busby, Jeremy Youde, and Asha Herten-Crabb, ‘From Imperialism to the. “Golden Age” to the Great Lockdown: The Politics of Global Health Governance’, https://eprints.lse.ac.uk/119772/1/From_Imperialism_to_the.pdf accessed 20 March 2025.

31 Randall M Packard [41]

32 Obichukwu Iwunna, Jonathan Kennedy and Andrew Harmer [42]

33 Marcos Cueto, Theodore M Brown and Elizabeth Fee [43] See also, Susan Gilbert [44]

34 David Fidler [45]

35 David Stuckler and Sanjay Basu [46]

36 Micheal Kofi Boachie, John Agyemang and Mustapha Immurana [47] See also, Global Policy Forum [48]

37 The World Trade Organization’s (WTO) Agreement on Trade-Related Aspects of Intellectual Property Rights (TRIPS), Article 33. See also, Access Campaign, ‘1998: Big Pharma versus Nelson Mandela’ (Médecins Sans Frontières Access Campaign) https://msfaccess.org/1998-big-pharma-versus-nelson-mandela accessed 17 March 2025. See also, Pat Sidley [49]

38 Tahir Amin and Aaron S Kesselheim, supra note 16.

39 Jianying Yue and others [50]

40 University of Washington [51] See also, Sakshi Prasad and others [52]

41 Eneasato Onyekachi [53]

42 Bambos Charalambous, ‘Low-income Countries: Debt Cancellation’, https://hansard.parliament.uk/commons/2025–02-06/debates/CAC6A479-E79E-4723-BE88-42C9AA65EEF6/Low-IncomeCountriesDebtCancellation accessed 20 March 2025.

43 Basic Principles and Guidelines on the Right to a Remedy and Reparation for Victims of Gross Violations of International Human Rights Law and Serious Violations of International Humanitarian Law, UNGA Resolution 60/147.

44 The United Nations Framework Convention on Climate Change (UNFCCC), 1992.
